# Supplementary material for: Capturing Multiple Timescales of Adaptation to Second-Order Statistics With Generalized Linear Models: Gain Scaling and Fractional Differentiation
Source: Front Syst Neurosci. 2020 Sep 9;14:60. doi: 10.3389/fnsys.2020.00060 (PMC7509073; doi:10.3389/fnsys.2020.00060)
Supplement: Supplementary file 1 [file Data_Sheet_1.PDF]

Supplementary Information: Capturing multiple timescales of adaptation to second-order statistics with generalized linear models: gain scaling and fractional differentiation

Authors: Kenneth W. Latimer [1,*] and Adrienne L. Fairhall [2]
[1]Department of Neurobiology, University of Chicago, Chicago, IL, USA
[2]Department of Physiology & Biophysics, University of Washington, Seattle, WA, USA
* Corresponding Author: latimerk@uchicago.edu

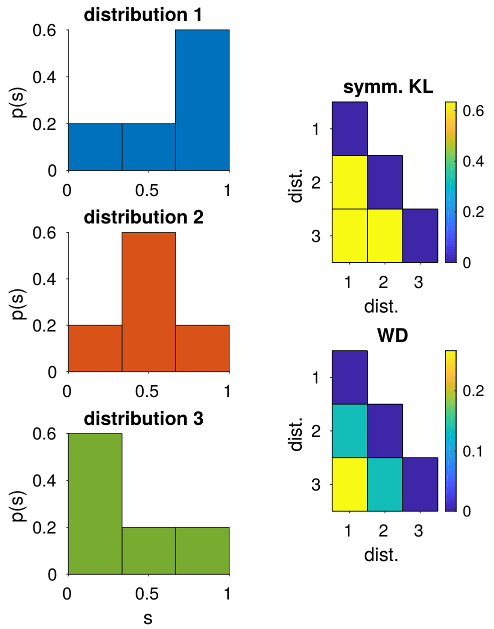

Supplementary Figure 1: Example differences between the Kullback-Leibler (KL) divergence with the Wasserstein distance used to measure gain scaling. The difference in the three distributions is probability mass moved along the axis. (Left) Three example distributions over the value s are given. (Right) The (symmetrized) KL divergence between each of the three distributions and the Wasserstein distances. The Wasserstein metric depends on the distance the peak of probability mass is moved along the axis: the distance between distributions 1 and 3 is greater than between distributions 1 and 2. In contrast, the KL divergence does not depend on the distance and the divergence between each pair of distributions is equal.

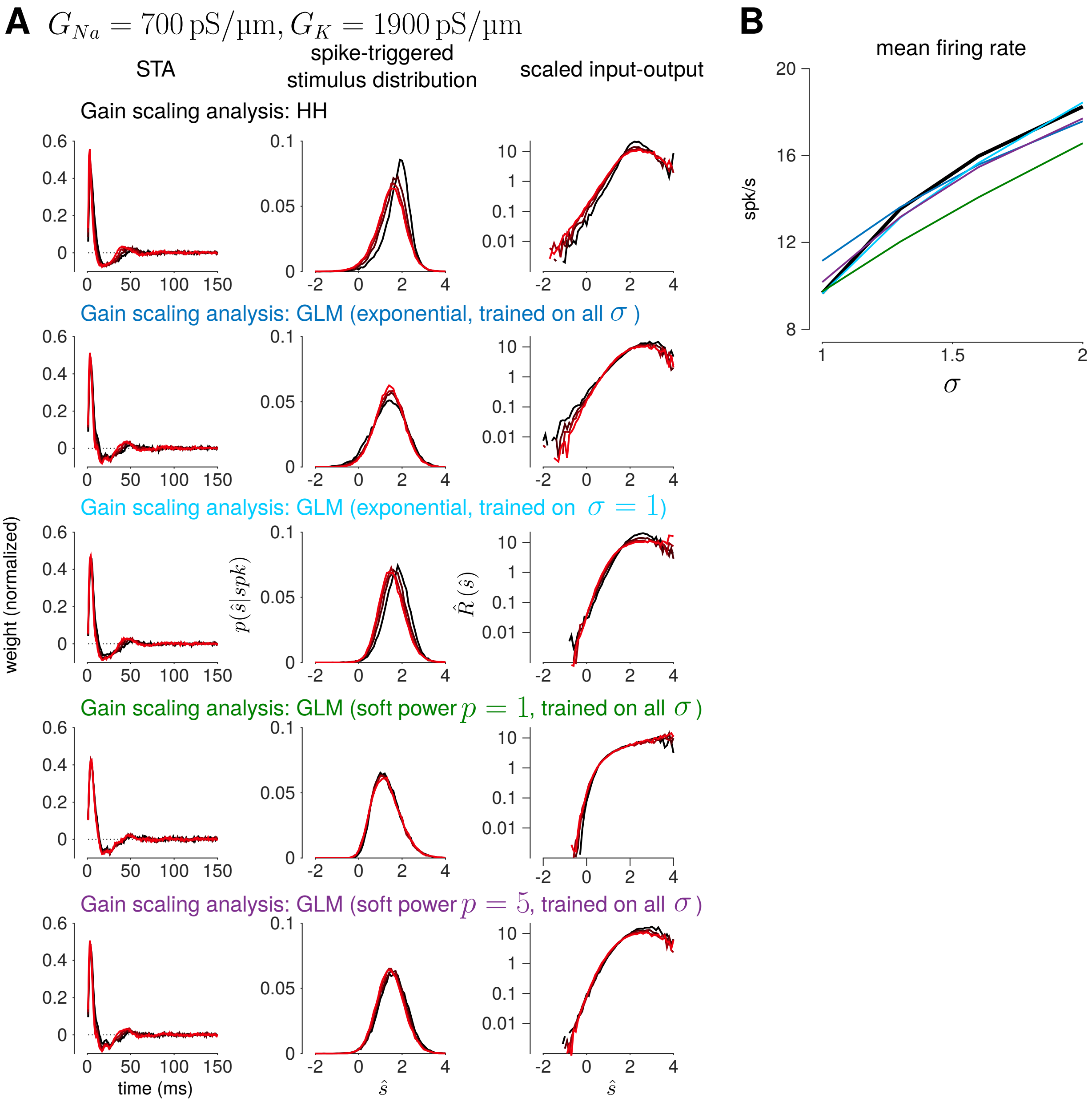

Supplementary Figure 2: (**A**) Example gain scaling analysis for four different GLM fits to the HH simulation in the top row. The HH simulation has a low sodium to potassium ratio with poor gain scaling. (**B**) The firing rates of the HH and GLM simulations as a function of σ.

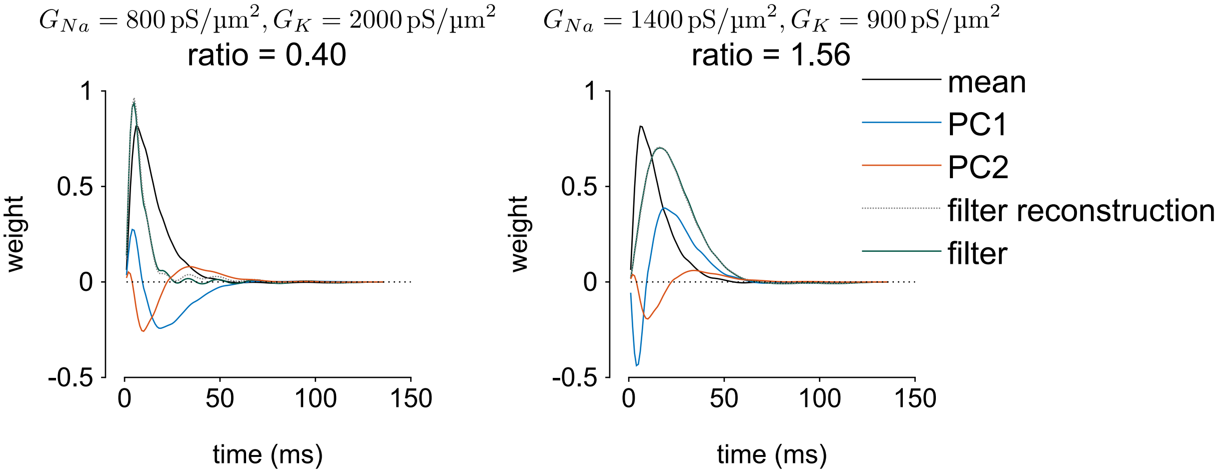

Supplementary Figure 3: Example PCA reconstruction of the GLM's stimulus filter for two of the HH fits. The mean filter and the two weighted PC vectors are given. The filter is reconstructed from the 2-D PCA space as the sum of the mean and the two PCs (dashed gray trace), and the reconstruction can be compared to the GLM filter (dark teal trace). Adding the weighted combinations of the two PCs extends or shortens the mean filter instead of adding multiple modes.
